# Association between the neutrophil-to-high-density lipoprotein cholesterol ratio with kidney stone risk: a cross-sectional study

**DOI:** 10.3389/fendo.2025.1523890

**Published:** 2025-02-03

**Authors:** Yuan-Zhuo Du, Jia-Qing Yang, Ji-Ming Yao, Chi-Teng Zhang, Yi-Fu Liu

**Affiliations:** ^1^ Department of Urology, The First Affiliated Hospital, Jiangxi Medical College, Nanchang University, Nanchang, China; ^2^ The Second Affiliated Hospital, Department of Urology, Hengyang Medical School, University of South China, Hengyang, Hunan, China

**Keywords:** neutrophil-to-high-density lipoprotein cholesterol ratio, kidney stones, neutrophil, high-density lipoprotein cholesterol, cross-sectional study

## Abstract

**Objective:**

Kidney stones are a major issue for public health worldwide. Discovering potential clues in identifying at-risk individuals is essential for early detection and timely treatment. This study explores the relationship of the neutrophil-to-high-density lipoprotein cholesterol ratio (NHR) with the risk of kidney stones in U.S. adults.

**Methods:**

The analysis involved 24,532 participants with available NHR and kidney stone data from the 2007–2018 NHANES period. Multivariable logistic regression models were used to quantify the relationship between NHR and kidney stone occurrence. Subgroup analyses were conducted to explore variations in effect.

**Results:**

A total of 2,351 participants (9.93%) were diagnosed with kidney stones, and their mean age was 47.20 ± 0.26 years. After full adjustment in the multivariable regression model, higher NHR levels were linked to a greater risk of kidney stones (OR = 1.05, 95% CI: 1.02–1.08, P = 0.002). Participants in the highest tertile of NHR had a 34% increased chance of kidney stone development compared to those in the lowest tertile. A nonlinear connection between NHR and kidney stone risk was identified using restricted cubic spline (RCS) regression models. The relationship between NHR and kidney stone prevalence showed no significant variation across most subgroups (P for interaction > 0.05).

**Conclusion:**

The results indicate that increased NHR is linked to a higher risk of kidney stones, with this relationship remaining consistent across various populations. NHR could be a useful biomarker for kidney stone risk, with key implications for early detection and individualized treatment.

## 1 Introduction

Kidney stones are a common urinary tract disorder and pose a significant global public health challenge (1, 2). The formation of kidney stones is associated with various factors, including genetic, environmental, and metabolic influences (3, 4). However, the complex pathophysiological mechanisms underlying kidney stone formation remain largely unclear. Modern lifestyle changes, such as shifts in dietary habits and reduced physical activity, have contributed to a rising incidence of kidney stones (5, 6). Therefore, early identification of high-risk individuals through biomarkers and effective prevention strategies is of great value to public health.

In recent years, biomarkers have played an increasingly important role in disease diagnosis and prognosis assessment. While several biomarkers, such as urinary calcium, oxalate, and uric acid, have been proposed for predicting kidney stone risk (7), their routine clinical application is limited due to the complex sampling and processing procedures required. Hence, there is an urgent need for simple yet effective predictive markers for early diagnosis and intervention. Neutrophils, as key players in the inflammatory process, typically increase in number when acute or chronic inflammation is present (8, 9). High-density lipoprotein cholesterol (HDL-C) is recognized as a cardiovascular protective factor attributed to its anti-inflammatory and antioxidant properties (10, 11). The neutrophil-to-high-density lipoprotein cholesterol ratio (NHR), a biomarker reflecting immune response and cholesterol balance, has shown significant clinical value in predicting cardiovascular diseases (12, 13).

Although the physiological mechanisms of kidney stone formation differ from those of cardiovascular disease, both conditions share inflammation and metabolic dysregulation as key factors. Inflammation promotes a favorable local environment for stone formation, while lipid metabolism abnormalities may directly impair the kidneys’ ability to handle minerals (14, 15). Therefore, this study, based on a large-scale cross-sectional analysis of U.S. adults from the National Health and Nutrition Examination Survey database, aims to evaluate whether NHR could serve as an effective biomarker for assessing kidney stone risk.

## 2 Methods

### 2.1 Study population

Data for this cross-sectional study were drawn from the 2007–2018 NHANES, which collects data biennially. The study received ethical approval from the Institutional Review Board of the National Center for Health Statistics, and all participants provided written informed consent. A qualified sample was chosen from 59,842 initial candidates, following set inclusion and exclusion guidelines. Excluded were those under 20 years old (n = 25,072), missing kidney stone questionnaire responses (n = 91), participants without complete NHR data (n = 3,495), and those missing important covariate details (n = 6,652). In total, 24,532 eligible participants were selected for the final analysis (**Supplementary Figure 1**).

### 2.2 Definition of NHR

NHR was calculated as the ratio of the neutrophil count (in units of 10^3 cells/μL) to the HDL-C level (in mmol/L). To ensure the accuracy of the complete blood count (CBC), the Beckman Coulter DxH 800 analyzer was employed. This system combines advanced counting technology with molecular mass detection, featuring automated sample dilution, mixing equipment, and a photometer for hemoglobin measurement. The NHANES mobile examination centers utilized this device to provide participants with detailed full blood count (FBC) reports and cell distribution data, guaranteeing accuracy and reliability in hematologic assessments. Enzyme assays were employed to accurately measure HDL cholesterol levels using the Roche Modular P and Cobas 6000 systems. All methods adhered to standardized procedures outlined in the NHANES CBC protocol, ensuring data accuracy and reproducibility.

### 2.3 Diagnosis of kidney stones

The study determined participants’ kidney stone history with the question, ‘Have you ever had kidney stones?’ This self-reported information has been shown to be reliable in previous studies (16). Those who responded ‘yes’ were categorized as having a history of kidney stones.

### 2.4 Definition of covariates

This research includes a variety of covariates associated with NHR and kidney stone risk, divided into three main categories: demographic indicators, lifestyle factors, and health status. Demographic indicators comprise age, gender, ethnicity, marital status, level of education, and poverty income ratios (PIR). Lifestyle factors cover alcohol consumption (categorized into lifelong abstainers who drank less than 12 times, former drinkers who drank 12 times or more but abstained in the past year, and current drinkers who have consumed alcohol 12 times or more in their lifetime and at least once in the last year) (18), smoking status (based on whether an individual has smoked over 100 cigarettes in their lifetime), sedentary behavior (daily sitting time exceeding 5 hours), and physical activity level (assessed by the duration of moderate to intense activity lasting at least 10 minutes per week beyond regular work and commuting, with less than 10 minutes classified as inactive) (17). Health indicators were collected using standardized questionnaires and clinical evaluations. Individual cell counts (1,000 cells/uL) for white blood cells, lymphocytes, monocytes, neutrophils, eosinophils, and basophils were obtained, along with biochemical markers such as total cholesterol (TC, mg/dL), triglycerides (TG, mmol/L), HDL-C (mmol/L), body mass index (BMI), estimated glomerular filtration rate (eGFR), and the presence of diabetes, hypertension, hyperlipidemia, and cardiovascular disease (CVD).

### 2.5 Statistical analysis

Statistical analyses incorporated NHANES’s sampling weights. Continuous variables are reported as weighted means and standard errors, while categorical variables are shown as weighted counts and proportions. Analyses involved weighted linear regression and chi-square tests. Multivariable logistic regression models were used to investigate the association between NHR and the incidence of kidney stones, providing odds ratios (ORs) and 95% confidence intervals (CIs). Models were stratified by covariates: unadjusted crude model; Model 1 adjusted for basic demographics; Model 2 further considered lifestyle and health status. Restricted cubic spline (RCS) regression models examined the dose-response relationship between NHR and kidney stone risk, with subgroup analyses for robustness. All analyses were performed using R software (version 4.3.2), with a significance level set at P<0.05.

## 3 Results

### 3.1 Baseline characteristics of participants

In this study, 24,532 participants from six NHANES cycles (2007-2018) were included, with an average age of 47.20 years (SE = 0.26 years), as presented in **Table 1**. Among these participants, 2,351 were diagnosed with kidney stones. The results indicated that participants in the kidney stone group were generally older, with a higher proportion of males and non-Hispanic whites. They were also more likely to be single (divorced/separated/widowed), had higher BMI, were more likely to be smokers or former drinkers, exercised less, had a lower eGFR within the normal range, and had higher levels of white blood cells, monocytes, neutrophils, and eosinophils. They also exhibited lower levels of HDL-C and higher TG levels. These participants also had a higher likelihood of a history of hypertension, diabetes, hyperlipidemia, and CVD. Furthermore, elevated NHR levels were associated with a greater risk of kidney stones.

**Table 1:** Baseline characteristics of the study population.

Variable	Overall (n = 24532)	Non-stone formers (n=22181)	Stone formers (n=2351)	P-value
Age, y, mean (SE)	47.20(0.26)	46.56(0.27)	52.96(0.38)	< 0.0001
Age strata, y, n (%)				< 0.0001
20–39	8327(36.68)	7863(38.37)	464(21.31)	
40–59	8163(37.78)	7346(37.26)	817(42.49)	
≥60	8042(25.55)	6972(24.37)	1070(36.20)	
Sex, n (%)				< 0.0001
Female	12447(51.14)	11418(51.85)	1029(44.76)	
Male	12085(48.86)	10763(48.15)	1322(55.24)	
Race, n (%)				< 0.0001
Mexican American	3623(8.17)	3324(8.42)	299(5.94)	
Non-Hispanic White	10748(69.01)	9428(67.97)	1320(78.40)	
Non-Hispanic Black	4930(10.14)	4628(10.64)	302(5.56)	
Other Hispanic	2462(5.38)	2206(5.42)	256(5.00)	
Other Race	2769(7.30)	2595(7.54)	174(5.10)	
Marital status, n (%)				< 0.0001
Divorced/Separated/Widowed	5382(18.06)	4764(17.74)	618(20.98)	
Married/Living with a partner	14696(63.91)	13186(63.28)	1510(69.63)	
Never married	4454(18.03)	4231(18.98)	223(9.38)	
Education levels, n (%)				0.45
High school and below	11148(37.20)	10053(37.10)	1095(38.11)	
Above high school	13384(62.80)	12128(62.90)	1256(61.89)	
PIR, n (%)				0.1
<1.3	7673(20.91)	6943(21.08)	730(19.40)	
1.3-3.5	9255(35.40)	8343(35.14)	912(37.75)	
>3.5	7604(43.69)	6895(43.78)	709(42.86)	
BMI, n (%)				< 0.0001
<18.5	361(1.44)	348(1.52)	13(0.71)	
18.5-24.99	6589(27.72)	6147(28.71)	442(18.72)	
25-29.99	8068(32.92)	7284(32.96)	784(32.60)	
≥30	9514(37.93)	8402(36.82)	1112(47.97)	
Smoke, n (%)				< 0.0001
No	13582(55.83)	12443(56.49)	1139(49.86)	
Yes	10950(44.17)	9738(43.51)	1212(50.14)	
Alcohol user, n (%)				< 0.0001
Never	3401(10.46)	3091(10.48)	310(10.26)	
Former	3835(12.66)	3337(12.16)	498(17.19)	
Now	17296(76.89)	15753(77.36)	1543(72.56)	
Moderate recreational activity, n (%)				< 0.0001
No	12533(44.77)	11155(44.00)	1378(51.70)	
Yes	11999(55.23)	11026(56.00)	973(48.30)	
Sitting time, n (%)				0.43
<5	9970(36.83)	9051(36.94)	919(35.78)	
≥5	14562(63.17)	13130(63.06)	1432(64.22)	
Hypertension, n (%)				< 0.0001
No	15713(68.26)	14550(69.91)	1163(53.32)	
Yes	8819(31.74)	7631(30.09)	1188(46.68)	
Diabetes, n (%)				< 0.0001
No	20830(88.42)	19075(89.46)	1755(78.91)	
Borderline	579(2.07)	496(1.92)	83(3.44)	
Yes	3123(9.51)	2610(8.62)	513(17.65)	
Hyperlipidemia, n (%)				< 0.0001
No	6971(29.50)	6491(30.44)	480(21.03)	
Yes	17561(70.50)	15690(69.56)	1871(78.97)	
CVD, n (%)				< 0.0001
No	23771(97.71)	21559(97.92)	2212(95.83)	
Yes	761(2.29)	622(2.08)	139(4.17)	
eGFR (mL/min), mean (SE)	94.64(0.34)	95.36(0.35)	88.07(0.52)	< 0.0001
White blood cell count (1000 cells/uL), mean (SE)	7.26(0.03)	7.24(0.03)	7.46(0.07)	0.002
Lymphocyte number (1000 cells/uL), mean (SE)	2.15(0.01)	2.15(0.01)	2.12(0.02)	0.21
Monocyte number (1000 cells/uL), mean (SE)	0.57(0.00)	0.56(0.00)	0.58(0.01)	0.004
Neutrophils number (1000 cell/uL), mean (SE)	4.31(0.02)	4.28(0.02)	4.50(0.06)	< 0.001
Eosinophils number (1000 cells/uL), mean (SE)	0.20(0.00)	0.20(0.00)	0.21(0.00)	0.003
Basophils number (1000 cells/uL), mean (SE)	0.05(0.00)	0.05(0.00)	0.05(0.00)	0.12
HDL-C (mmol/L), mean (SE)	1.38(0.01)	1.39(0.01)	1.29(0.01)	< 0.0001
TG (mmol/L), mean (SE)	1.72(0.02)	1.70(0.02)	1.95(0.07)	< 0.001
TC (mg/dL), mean (SE)	193.61(0.53)	193.70(0.53)	192.84(1.26)	0.48
NHR, mean (SE)	3.47(0.03)	3.42(0.03)	3.88(0.07)	< 0.0001

NHR, neutrophil-to-high-density lipoprotein cholesterol ratio; eGFR, estimated glomerular filtration rate; PIR, Poverty income ratio; BMI, body mass index; CVD, cardiovascular disease; HDL-C, high-density lipoprotein cholesterol; TG, triglycerides; TC, total cholesterol.

### 3.2 Association between NHR and kidney stones

**Figure 1:**
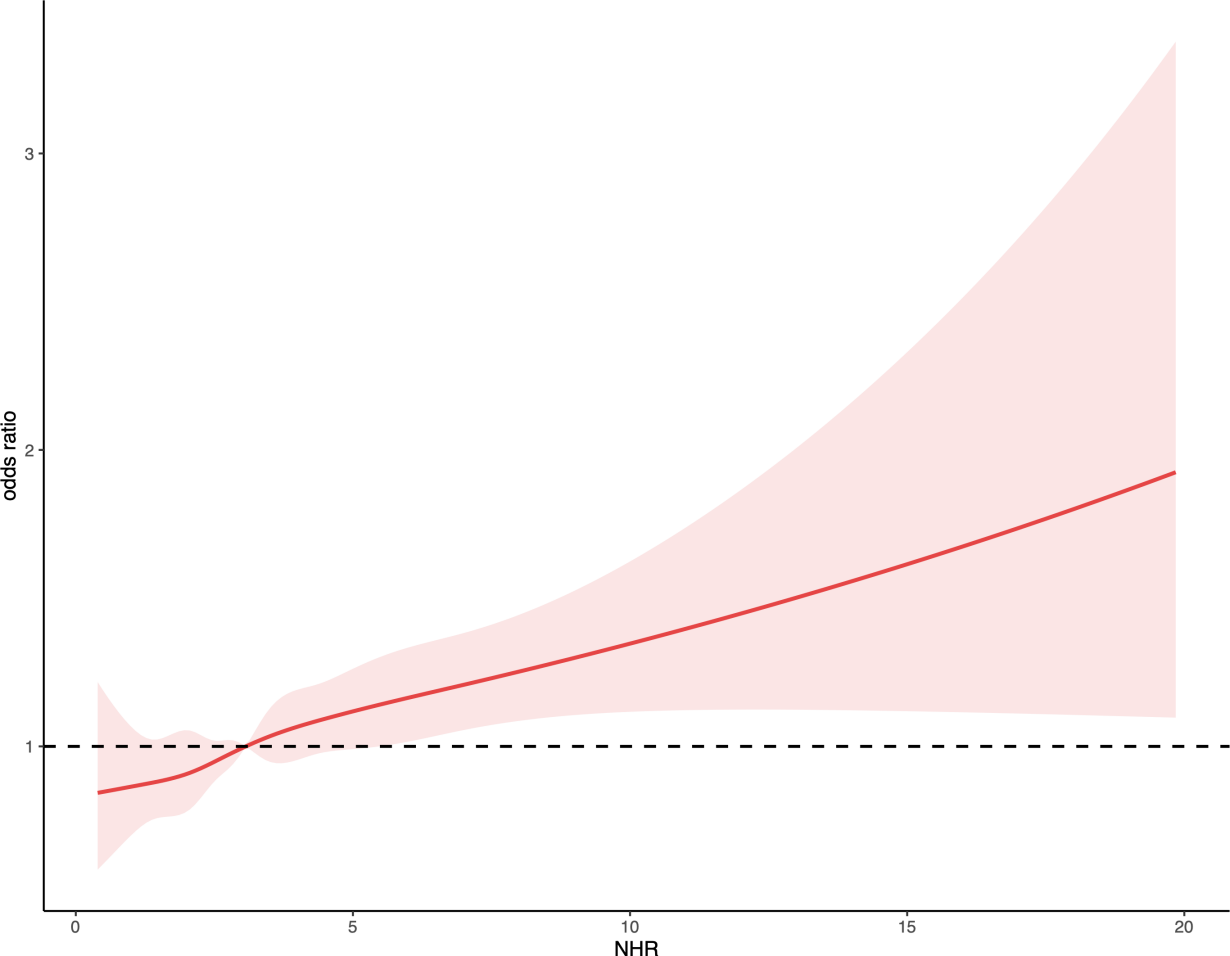
Depicts the association between NHR and kidney stone occurrences. The ORs, shown as solid lines, were adjusted for factors including age, gender, ethnicity, marital status, educational attainment, PIR, BMI, smoking habits, alcohol consumption, recreational activities, sitting duration, eGFR, hypertension, diabetes, hyperlipidemia, and CVD. The corresponding 95% CIs are indicated by shaded regions.

**Table 2** shows the relationship between NHR and kidney stones as analyzed through logistic regression models. In the unadjusted model, an increase of one unit in NHR was linked to an 11% rise in kidney stone risk (95% CI: 1.09-1.14, P < 0.0001). This significant positive association persisted even after adjusting for all covariates in Model 2, where each unit increase in NHR was linked to a 5% increase in the risk of kidney stones (95% CI: 1.02-1.08, P = 0.002). Additionally, those in the highest tertile of NHR had a 34% greater risk of developing kidney stones compared to participants in the lowest tertile (95% CI: 1.15-1.57, P < 0.001). The RCS regression, adjusted for relevant covariates, indicated a significant nonlinear association between NHR and kidney stone risk (nonlinear P < 0.05). A gradual increase in kidney stone risk was observed with higher NHR levels (**Figure 1**).

**Table 2:** Association of the tertiles of NHR with kidney stone.

Exposure	Crude model		Model 1		Model 2	
	OR (95% CI)	P value	OR (95% CI)	P value	OR (95% CI)	P value
NHR	1.11(1.09,1.14)	<0.0001	1.11(1.08,1.14)	<0.0001	1.05(1.02,1.08)	0.002
NHR Tertiles						
Tertile 1 (≤2.44)	1 (Ref.)		1 (Ref.)		1 (Ref.)	
Tertile 2 (2.45–3.84)	1.29(1.10,1.51)	0.002	1.25(1.07,1.46)	0.01	1.10(0.93,1.29)	0.25
Tertile 3 (≥3.85)	1.82(1.59,2.08)	<0.0001	1.75(1.53,2.00)	<0.0001	1.34(1.15,1.57)	<0.001
P for trend		<0.0001		<0.0001		<0.001

Crude model: unadjusted model;

Model 1: Adjusted for age, sex, race, education levels, marital status, PIR;

Model 2: Additionally adjusted for BMI, smoking, alcohol user, recreational activity, sitting time, eGFR, hypertension, diabetes, hyperlipidemia and CVD.

NHR, neutrophil to high-density lipoprotein cholesterol ratio; OR, odds ratio; CI, confidence interval.

### 3.3 Subgroup analyses

**Figure 2:**
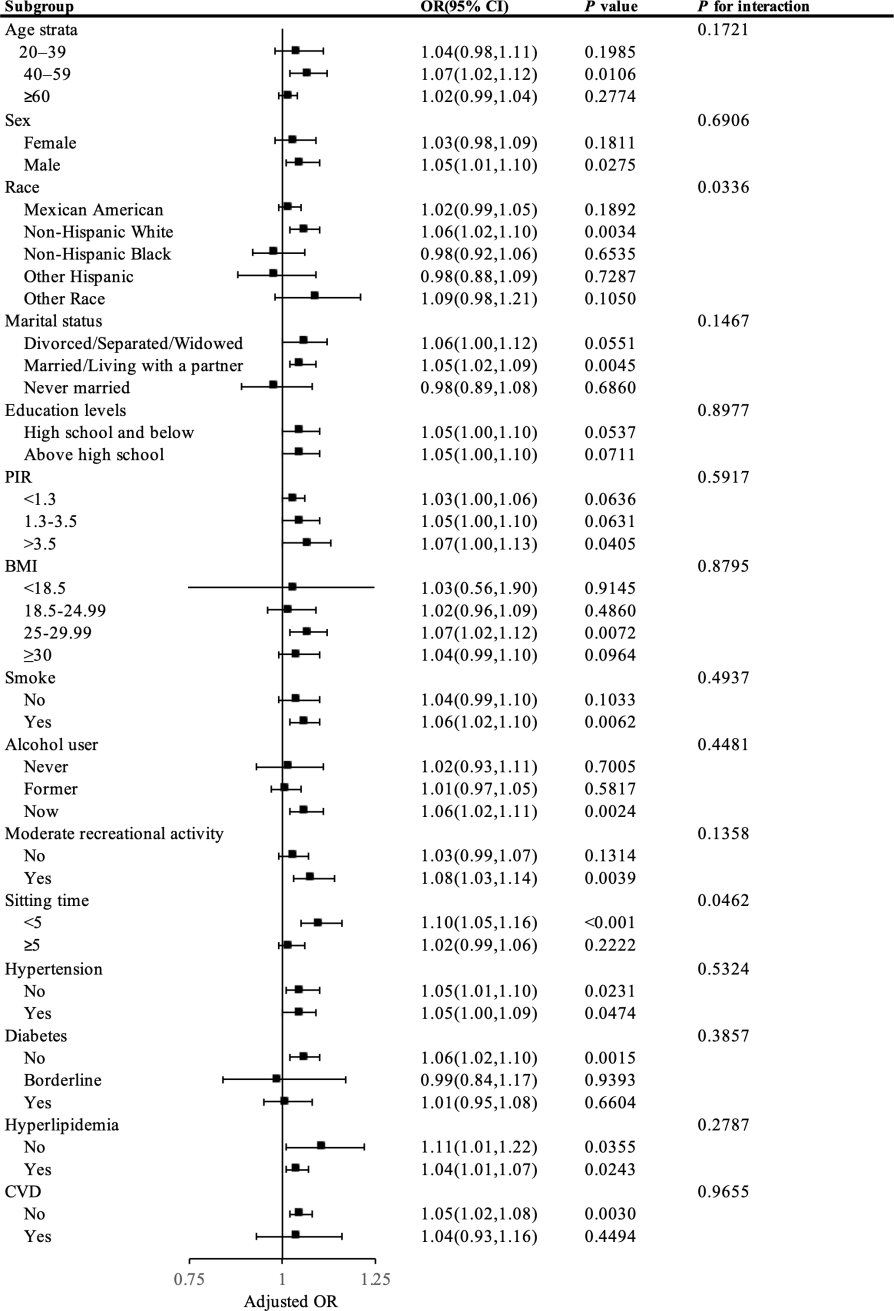
Presents a stratified analysis of NHR and kidney stone.

**Figure 2** demonstrates that the stratified analysis uncovered differences in the link between NHR and kidney stone risk across various subpopulations. In the fully adjusted multivariable model (excluding the stratification factor itself), the association between NHR and kidney stones did not significantly differ across most subgroups (P-values for interactions > 0.05).

## 4 Discussion

This study explored the connection between the NHR and kidney stone risk, utilizing data from the NHANES database. Our findings indicate that an elevated NHR is significantly linked to an increased risk of kidney stones, underscoring the importance of NHR as a biomarker that reflects both inflammation and lipid metabolism dysregulation. This suggests that the NHR may have potential clinical utility in assessing the likelihood of developing kidney stones.

Neutrophils, as key players in the immune response, often signal increased inflammatory activity when elevated in number formation, inflammatory processes may directly impact renal health by promoting cholesterol crystal deposition (18–20). Neutrophils release oxidative and inflammatory mediators, such as myeloperoxidase, which not only damage renal epithelial cells but also promote crystal formation and growth in urine, further increasing the risk of kidney stones (21–23). HDL-C is involved in reverse cholesterol transport, reducing cholesterol accumulation in the vascular and renal systems (24, 25), while also exerting anti-inflammatory and antioxidant effects (26, 27). A decline in HDL-C may lead to local lipid accumulation and increased oxidative stress in kidney stones (28, 29).

NHR is more than just a marker of inflammation; it is also an important indicator of lipid metabolism abnormalities. The formation of kidney stones is linked to disorders, particularly inflammation resulting from dysregulation of lipid metabolism (4, 30). Changes in the relative proportions of neutrophils and HDL-C reflect the interplay between these two biological activities, which not only directly damage renal tissue but also affect oxidative stress and inflammation in the renal microenvironment, increasing the likelihood of stone formation. Simultaneously, lower HDL-C reduces the body’s ability to clear oxidants, diminishing its protective effects on the kidneys, which may further promote kidney stone formation.

In this study, we explored the association between the NHR and kidney stone risk, particularly analyzing how this relationship manifests in individuals across different BMI categories. Notably, our findings indicate that NHR does not confer a protective effect against kidney stones in individuals with a BMI greater than 30. This suggests that the metabolic disturbances typically associated with obesity—such as enhanced inflammation and insulin resistance—may attenuate the potential protective effects encapsulated by the HDL component within the NHR. To thoroughly assess the impact of obesity on NHR and kidney stone risk, we stratified the study population into four BMI categories: underweight (<18.5), normal weight (18.5-24.99), overweight (25-29.99), and obese (≥30). This classification is grounded in widely recognized clinical and epidemiological standards, facilitating a nuanced analysis of how obesity modifies the effects of NHR on kidney stone risk. Each category reflects unique metabolic and physiological characteristics that can significantly influence the interplay between neutrophil counts, HDL levels, and the subsequent risk of kidney stone formation.

Further elucidating the role of NHR in different health conditions, our findings revealed distinct associations with kidney stone risk across various health subgroups, particularly in individuals with hypertension and diabetes. For individuals with hypertension, a consistent odds ratio of approximately 1.05 in both hypertensive and non-hypertensive groups suggests that elevated NHR levels contribute to kidney stone risk independently of an individual’s hypertension status. This indicates that the pro-inflammatory and pro-oxidative conditions indicated by high NHR may enhance kidney stone risk through mechanisms that extend beyond hypertension-associated physiological changes. Conversely, the diabetes subgroup presented a more complex scenario. Non-diabetic individuals exhibited a significant increase in kidney stone risk (OR=1.06) associated with higher NHR, whereas those with active or borderline diabetes did not. This pattern suggests that the metabolic management strategies typically employed in diabetic care, such as glycemic control and lifestyle interventions, may effectively mitigate the risk factors for kidney stones that are exacerbated by higher NHR levels. These observations advocate for personalized medical approaches, particularly the precise monitoring and management of NHR in the context of diabetes, which could be tailored to leverage protective mechanisms unique to diabetic patients’ metabolic profiles.

From a clinical perspective, monitoring changes in NHR provides clinicians with an effective tool to predict and manage the risk of kidney stones. Frequent monitoring and early intervention in patients with high NHR levels may help reduce the incidence of kidney stones. Moreover, lifestyle modifications such as increased water intake, reduced salt and protein consumption, and appropriate pharmacological interventions can help regulate NHR levels and, in turn, control or lower the risk of kidney stones.

This study’s main strength is the use of NHR as a composite biomarker to examine the risk factors and mechanisms involved in kidney stone formation. NHR, which combines neutrophil count and HDL-C levels, offers a single indicator that reflects both inflammatory status and lipid metabolism health. This approach allows for a more comprehensive risk assessment by considering the interaction of these two biological processes within the same model, potentially offering a more holistic view than evaluating each factor separately. Additionally, the large-scale NHANES dataset increases the generalizability and statistical power of the results, making them more representative and broadly applicable.

However, this study has some limitations. Primarily, as a cross-sectional design, it is difficult to infer causal relationships. We cannot definitively conclude whether high NHR leads to kidney stone formation or if it is merely a common phenomenon among kidney stone patients. Although NHR provides a combined perspective on inflammation and lipid metabolism, it may not capture the full complexity of biological processes influencing kidney stone risk. For instance, NHR does not account for other important biomarkers related to stone formation, such as urinary calcium and oxalate. Lastly, the study did not account for all potential confounders, such as genetic predisposition and dietary habits, which could also influence the formation of kidney stones.

## 5 Conclusions

This research, grounded in the NHANES database, examined how the NHR correlates with the risk of kidney stones among American adults. The results showed a significant correlation between increased NHR and a higher risk of kidney stones. This finding suggests that NHR could be an effective tool for assessing kidney stone risk, potentially aiding in early diagnosis and preventive interventions.

## Data Availability

Publicly available datasets were analyzed in this study. This data can be found here: https://www.cdc.gov/nchs/nhanes/index.htm.
