# Supplementary material for: Association between the neutrophil-to-high-density lipoprotein cholesterol ratio with kidney stone risk: a cross-sectional study
Source: Front Endocrinol (Lausanne). 2025 Feb 3;16:1523890. doi: 10.3389/fendo.2025.1523890 (PMC11830614; doi:10.3389/fendo.2025.1523890)
Supplement: Supplementary file 1 [file DataSheet1.docx]

**Supplementary Material**

**Association between the neutrophil-to-high-density lipoprotein cholesterol ratio with kidney stone risk: a cross-sectional study**

Yuan-Zhuo Du ^1#^, Jia-Qing Yang ^1#^, Ji-Ming Yao ^1#^, Chi-Teng Zhang ^2*^, Yi-Fu Liu ^2*^

^#^ Co-first author：Yuan-Zhuo Du^1#^, Jia-Qing Yang^1#^, Ji-Ming Yao^1#^

^1^Department of Urology, The First Affiliated Hospital, Jiangxi Medical College, Nanchang University, Nanchang, China

* Correspondence: Chi-Teng Zhang ^2*^, Yi-Fu Liu ^2*^

^2^The Second Affiliated Hospital, Department of Urology, Hengyang Medical School, University of South China, Hengyang, Hunan, China

Chi-Teng Zhang ^2*^ Email: zct00523760@163.com

Yi-Fu Liu ^2*^ Email: lyf137556057762022@163.com

**Supplementary Figure 1:** The participant flow diagram.
